# Plasma Pattern of Extracellular Vesicles Isolated from Hepatitis C Virus Patients and Their Effects on Human Vascular Endothelial Cells

**DOI:** 10.3390/ijms241210197

**Published:** 2023-06-15

**Authors:** Elena Grossini, Carlo Smirne, Sakthipriyan Venkatesan, Stelvio Tonello, Davide D’Onghia, Rosalba Minisini, Vincenzo Cantaluppi, Pier Paolo Sainaghi, Cristoforo Comi, Adele Tanzi, Benedetta Bussolati, Mario Pirisi

**Affiliations:** 1 Laboratory of Physiology, Department of Translational Medicine, Università del Piemonte Orientale, 28100 Novara, Italy; sakthipriyan.venkatesan@uniupo.it; 2 Internal Medicine Unit, Department of Translational Medicine, Università del Piemonte Orientale, 28100 Novara, Italy; carlo.smirne@med.uniupo.it (C.S.); stelvio.tonello@med.uniupo.it (S.T.); davide.donghia@uniupo.it (D.D.); rosalba.minisini@med.uniupo.it (R.M.); pierpaolo.sainaghi@med.uniupo.it (P.P.S.); mario.pirisi@med.uniupo.it (M.P.); 3 Maggiore della Carità Hospital, 28100 Novara, Italy; 4 Nephrology Unit, Department of Translational Medicine, Università del Piemonte Orientale, 28100 Novara, Italy; vincenzo.cantaluppi@med.uniupo.it; 5 CAAD—Center for Autoimmune and Allergic Diseases, and IRCAD—Interdisciplinary Research Center for Autoimmune Diseases, Università del Piemonte Orientale, 28100 Novara, Italy; 6 Neurology Unit, Department of Translational Medicine, Università del Piemonte Orientale, 28100 Novara, Italy; cristoforo.comi@med.uniupo.it; 7 Sant’Andrea Hospital, 13100 Vercelli, Italy; 8 Molecular Biotechnology Center “Guido Tarone”, Department of Molecular Biotechnology and Health Sciences, University of Torino, 10124 Turin, Italy; adele.tanzi@unito.it (A.T.); benedetta.bussolati@unito.it (B.B.)

**Keywords:** cardiovascular disease, cell survival, endothelial dysfunction, exosomes, hepatitis C virus, hepatocellular carcinoma, liver cirrhosis, liver fibrosis, oxidative stress, vesicles

## Abstract

Hepatitis C virus (HCV) patients are at increased risk of cardiovascular disease (CVD). In this study, we aimed to evaluate the role of extracellular vesicles (EVs) as pathogenic factors for the onset of HCV-related endothelial dysfunction. Sixty-five patients with various stages of HCV-related chronic liver disease were enrolled in this case series. Plasma EVs were characterized and used to stimulate human vascular endothelial cells (HUVEC), which were examined for cell viability, mitochondrial membrane potential, and reactive oxygen species (ROS) release. The results showed that EVs from HCV patients were mainly of endothelial and lymphocyte origin. Moreover, EVs were able to reduce cell viability and mitochondrial membrane potential of HUVEC, while increasing ROS release. Those harmful effects were reduced by the pretreatment of HUVEC with the NLR family pyrin domain containing 3 (NLRP3)/AMP-activated protein kinase and protein kinase B blockers. In conclusion, in HCV patients, we could highlight a circulating pattern of EVs capable of inducing damage to the endothelium. These data represent a novel possible pathogenic mechanism underlying the reported increase of CVD occurrence in HCV infection and could be of clinical relevance also in relation to the widespread use of antiviral drugs.

## 1. Introduction

Emerging evidence suggests that chronic hepatitis C (HCV) infection is a systemic condition characterized by various immune-related disorders, metabolic (glucose and lipid) alterations, neuropsychiatric, kidney, and cardiovascular diseases (CVD) [1,2,3].

The chronicity of HCV infection, as well as the presence of the virus in non-hepatic tissues, may create a favorable milieu for the development of potential pathogenic impact on extrahepatic systems and organs, which can be found in up to 50% of infected patients [4].

In particular, the association between HCV infection and a higher incidence of major adverse cardiovascular events—such as coronary artery disease, heart failure, stroke, and peripheral artery disease—have been recently highlighted [3].

Previous observations lead to the hypothesis that metabolic syndrome could be involved as a possible mechanism of this increased cardiovascular risk. Hence, HCV could modulate lipid metabolism and insulin signaling, leading to a higher prevalence of diabetes and steatosis [5,6], and also potentiate atherosclerosis [7]. 

However, although metabolic factors may play a role [8], systemic chronic inflammation seems more likely to act as a central factor for HCV-related cardiovascular alterations [9,10]. Indeed, there is evidence of chronic inflammation induced by innate, natural killer cell (NK)-mediated, and adaptive T-helper-1-mediated responses [11] in patients infected with HCV. Hepatic steatosis and visceral obesity could additionally contribute to this inflammatory status by prompting the production of reactive oxygen species (ROS) and inflammatory cytokines [12], as well.

Along this line, endothelial damage directly and indirectly related to HCV infection [13,14] could further contribute to the link between hepatitis and the aforementioned higher cardiovascular risk through reduced nitric oxide (NO) release, increased platelet and leukocyte adhesion, pro-inflammatory cytokines production, and increased permeability for oxidized lipoproteins and plaque formation [7,15]. 

Although much is known about CVD and HCV disease, studies aimed at identifying the precise role of the virus in the onset of CVD are needed. In particular, it could be interesting to analyze the mechanisms responsible for cardiovascular alterations both in individuals with chronic active HCV infection and in those who achieved a sustained viral response after treatment with direct-acting antiviral (DAA) agents. In the former, in addition to a direct pathogenic effect of the virus, it is conceivable to hypothesize the presence of circulating factors, released in response to the infection by different tissues, and capable of maintaining and perpetuating cardiovascular damage even beyond the acute event [16]. In the latter, a significant early reduction of CVD incidence risk has been demonstrated, even if the mechanisms responsible are not yet fully understood, we still lack validated biomarkers to estimate the residual risk after virological recovery [2]. About this issue, promising candidates could be extracellular vesicles (EVs), which have been proven to transmit hepatitis C and to contribute to viral spread [2,5,17], in addition to providing a feedback loop to either exacerbate or reduce infection, inflammation, and endothelial dysfunction in a sort of systemic process [17,18,19].

Therefore, since EVs are increasingly considered important mediators and potential biomarkers of systemic inflammation, their measurement in biological fluids might be used in diagnostic algorithms and used for the detection and determination of the severity of diseases associated with inflammatory responses, such as HCV infection, as well as for the prediction of their outcome [20].

Based on the above, we hypothesized that in HCV subjects, circulating EVs may represent a mechanism of endothelial dysfunction, occurring along all disease stages, and contributing over the years to significant cardiovascular damage. To verify this hypothesis, we analyzed the plasma EVs pattern in HCV patients with different disease stages from mild hepatitis to hepatocellular carcinoma (HCC), and their effect on endothelial function. Furthermore, we also focused on the analysis of some of the most at present plausible intracellular pathways involved in these processes.

## 2. Results

As shown in Table 1, our study population was well representative of patients currently attending a liver clinic in a Western country [21]. More in detail, all patients were Caucasian, HCV mono-infected with native livers. Hepatitis C virus-RNA levels were measured using ABBOTT RealTime HCV assay with a lower limit of quantitation (LLQ) of 12 IU/mL and a lower limit of detection of 10 IU/mL (Abbott Laboratories; Abbott Park, IL, USA). The liver fibrosis stage was defined by histology and/or transient elastography, according to METAVIR classification [1]. Due to the intended balancing between all the different stages of liver disease, there was an obvious overrepresentation of the more advanced stages of fibrosis (and, consequently, of HCC) compared to what was expected *a priori* in a non-selected outpatient population (which would be around 30%) [22]. As reported in every HCV population, we found a high prevalence of metabolic diseases (such as diabetes, hypertension, or dyslipidemia) [23].

### 2.1. Characterization of Circulating EVs in HCV Patients

The EVs isolated from plasma of both HCV patients and healthy controls were characterized both in terms of concentration, size and surface marker expression.

**Figure 1:**
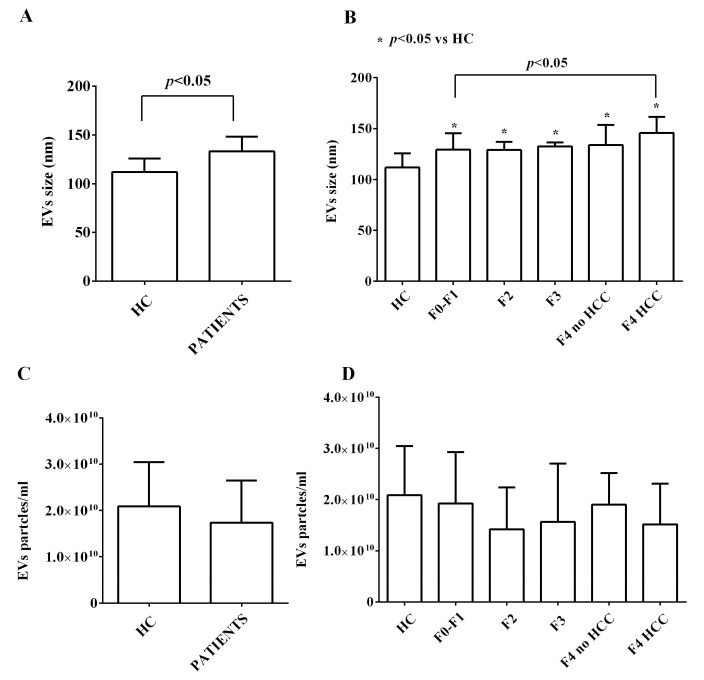
NanoSight analysis of extracellular vesicles size (**A**,**B**) and concentrations (**C**,**D**). The values are the means ± SD of repeated measurements. HC: healthy controls. HCC: hepatocarcinoma. F1, F2, F3, F4: METAVIR HCV stages. No HCC: cirrhosis without HCC. HCC: cirrhosis with HCC. Square brackets indicate significance between groups.

The NanoSight analysis showed that the size of plasma EVs of HCV patients was greater than that of the healthy controls (Figure 1A,B). In addition, we found that EVs of cirrhotic (F4) HCC patients had higher size than those with F0-F1 stages (Figure 1B). Instead, no differences were observed between HCV patients and healthy controls as regarding the concentration of EVs (Figure 1C,D). These results are in agreement with previous reports showing that EVs sizes rather than EVs concentrations are increased in liver failure patients and correlate with prognosis [24,25,26,27].

**Figure 2:**
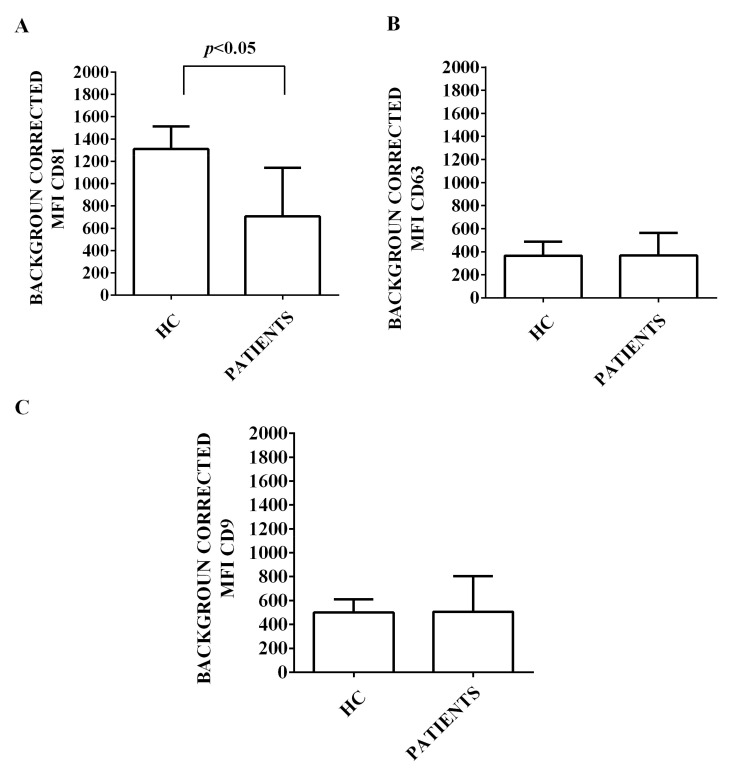
Results of MASPlex analysis. In (**A**–**C**), expression of exosomal markers by EVs isolated from HCV patients and healthy controls, is shown. The results represent background corrected CD9/CD63/CD81 MFI values normalized to the MFI of all detectable markers. Data are expressed as means ± SD. MFI: mean fluorescence intensity. HC: healthy controls. Square bracket indicates significance between groups.

**Figure 3:**
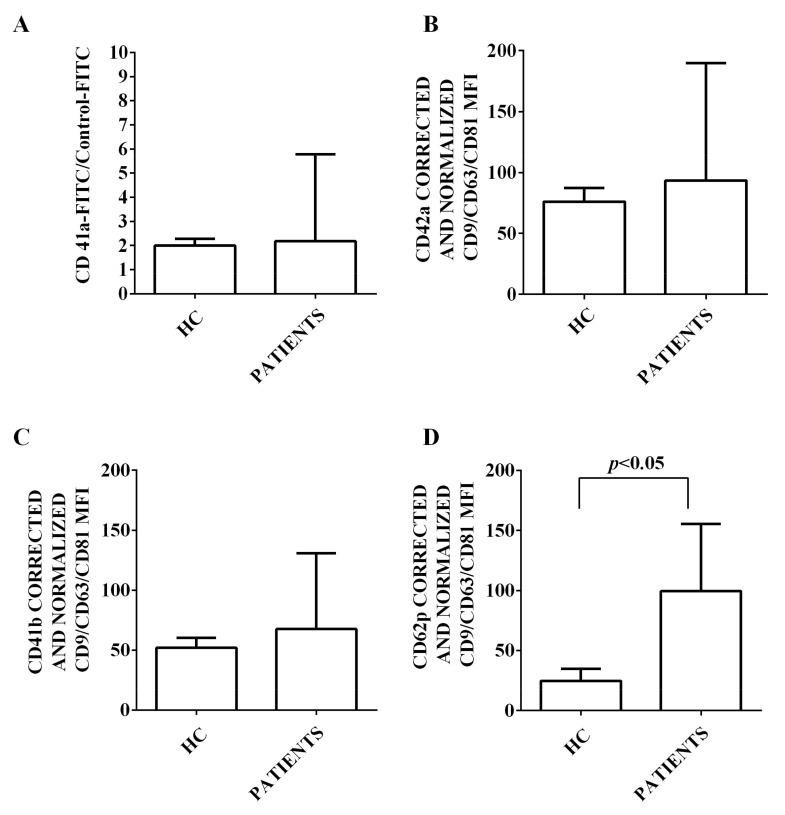
Expression of markers of platelet origin by EVs isolated from HCV patients and healthy controls. In (**A**), FACS analysis of CD41a expression. In (**B**–**D**), fluorescence intensity of CD42a, CD41b, and CD62p obtained by MACSPlex analysis. The intensity level of each marker was normalized to the MFI of all detectable markers and expressed as means ± SD. MFI: mean fluorescence intensity. HC: healthy controls. Square brackets indicate significance between groups.

**Figure 4:**
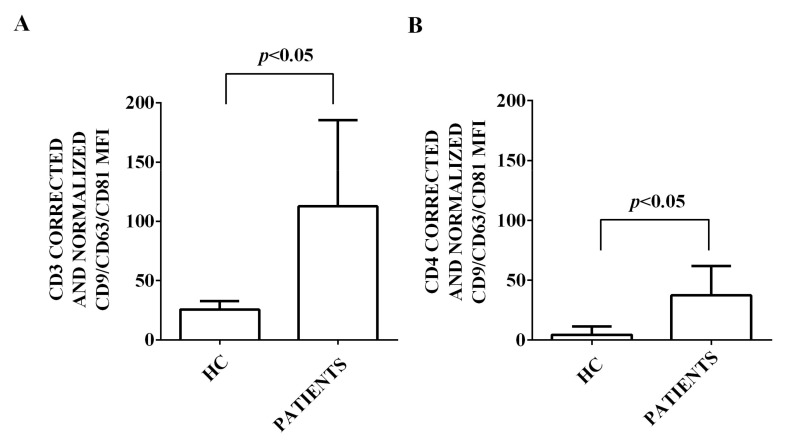

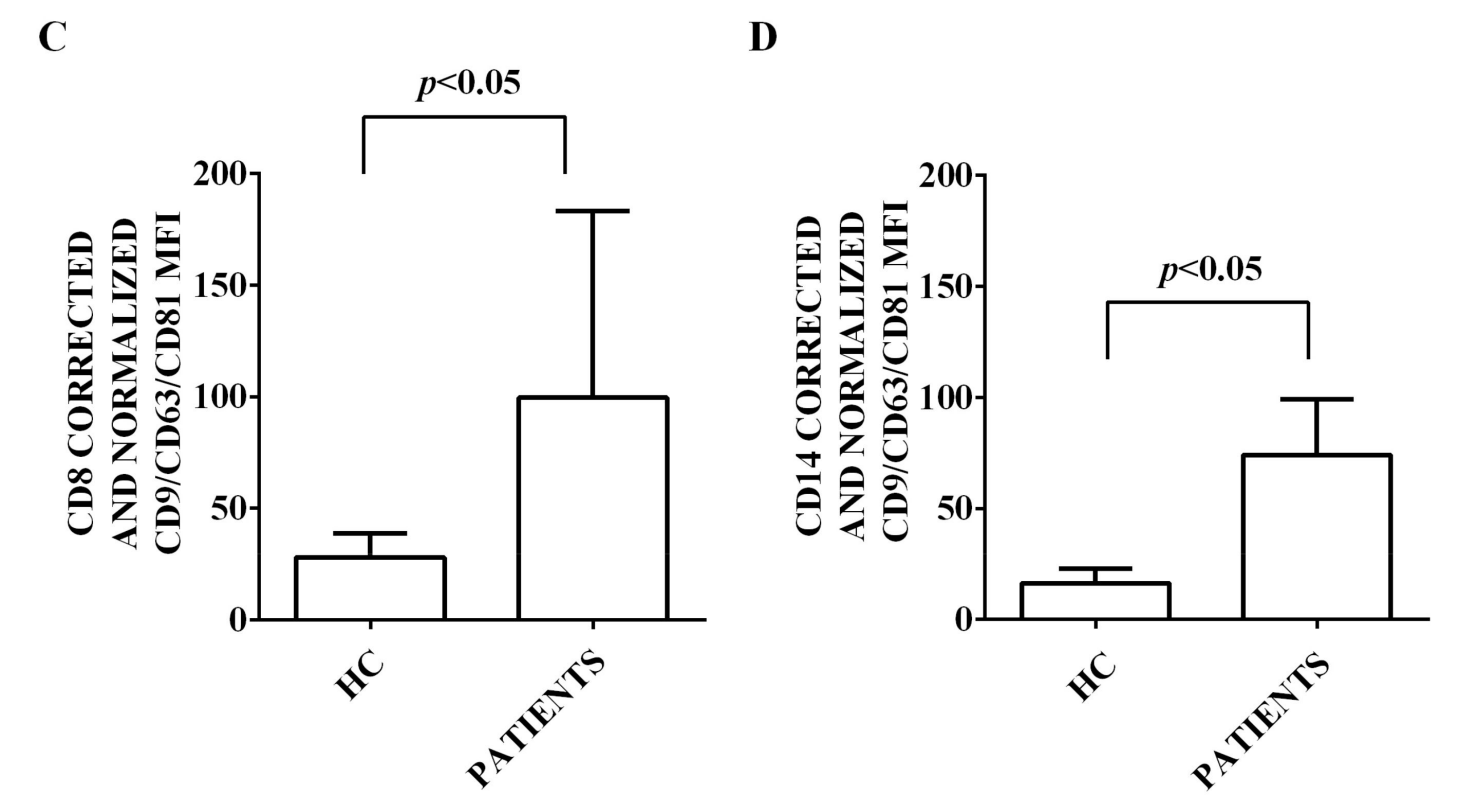
Expression of lymphocytic markers by EVs isolated from HCV patients and healthy controls. In (**A**), fluorescence intensity of CD3. In (**B**), fluorescence intensity of CD4. In (**C**), fluorescence intensity of CD8. In (**D**), fluorescence intensity of CD14. The intensity level of each marker was normalized to the MFI of all detectable markers and expressed as means ± SD. MFI: mean fluorescence intensity. HC: healthy controls. Square brackets indicate significance between groups.

**Figure 5:**
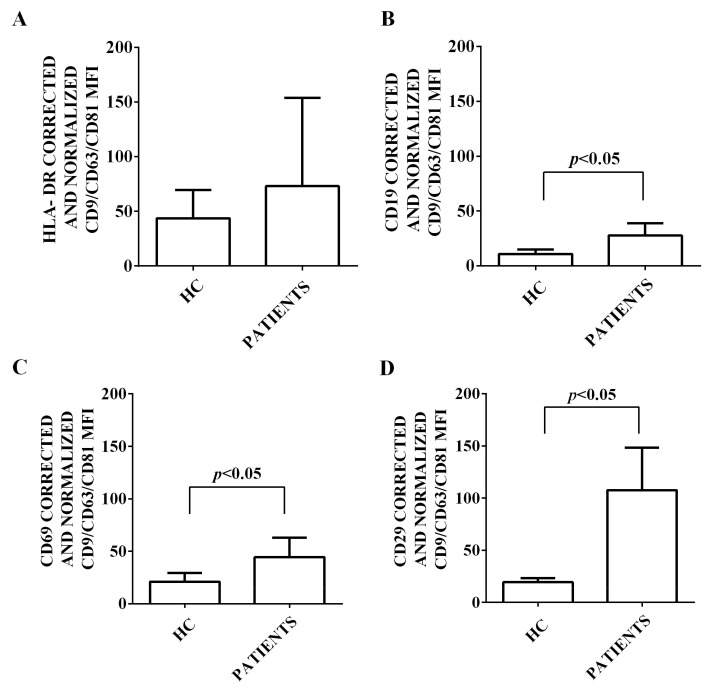
Expression of hematopoietic markers by EVs isolated from HCV patients and healthy controls. In (**A**), fluorescence intensity of HLA. In (**B**), fluorescence intensity of CD19. In (**C**), fluorescence intensity of CD69. In (**D**), fluorescence intensity of CD29. The intensity level of each marker was normalized to the MFI of all detectable markers and expressed as means ± SD. MFI: mean fluorescence intensity. HC: healthy controls. Square brackets indicate significance between groups.

**Figure 6:**
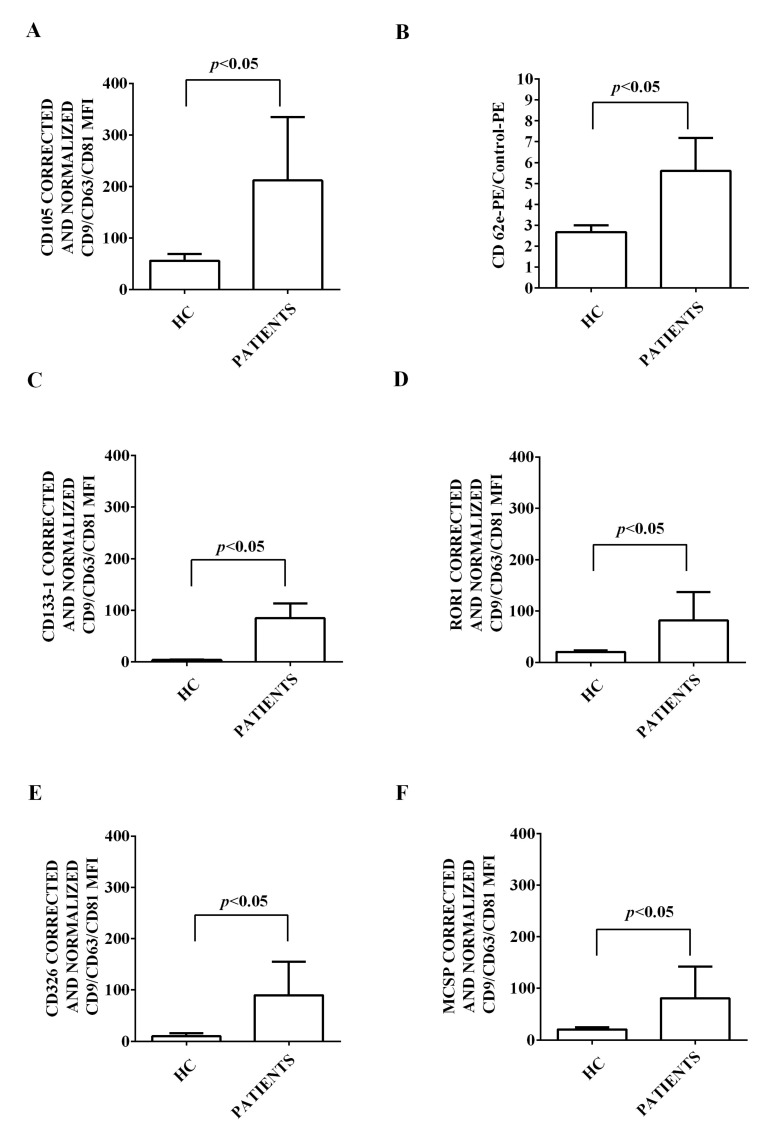
Expression of endothelial/regenerative/markers by EVs isolated from HCV patients and healthy controls. In (**A**), fluorescence intensity of CD105. In (**B**), fluorescence intensity of CD62e. In (**C**), fluorescence intensity of CD133-1. In (**D**), fluorescence intensity of ROR1. In (**E**), fluorescence intensity of CD326. In (**F**), fluorescence intensity of MCSP. The intensity level of each marker was normalized to MFI of all detectable markers and expressed as means ± SD. MFI: mean fluorescence intensity. HC: healthy controls. Square brackets indicate significance between groups (*p* < 0.05).

Moreover, we characterized the expression of 37 surface antigens in EVs isolated from the plasma of both HCV patients and healthy controls. This characterization has been performed by flow cytometry and using the MACSPlex Exosome kit. The results obtained are shown in Figure 2, Figure 3, Figure 4, Figure 5 and Figure 6. First of all, EVs have been shown to be positive for the typical exosome markers, CD9, CD63, and CD81 (Figure 2A–C). Moreover, all the plasma-EV samples resulted positive for the surface markers typical of various cell types such as platelet (CD42a, CD41b, and CD62p; Figure 3A–D) and hematopoietic cells (CD3, CD4, CD8, CD14, HLA-DR, DC19, CD69, and CD29; Figure 4A–D and Figure 5A–D). In addition, among endothelial markers, we could detect the expression of CD105 and CD62e (Figure 6A,B). Plasma-EVs samples expressed also epithelial adhesion molecules and the hematopoietic staminal mesenchymal ones (CD133-1, ROR1, CD326, and MCSP; Figure 6C–F) [28,29]. It is also to note that as regarding CD62p, CD3, CD4, CD8, CD14, CD19, CD69, CD29, CD105, and CD62e, the measured fluorescence intensity in EVs isolated from HCV patients was higher than in healthy controls (Figure 3, Figure 4, Figure 5 and Figure 6).

Other surface antigens, such as CD49e, CD142, CD146, SSEA-4, CD31, CD24, CD1, CD11c, CD2, CD25, CD56, CD209, CD45, CD20, and CD40, were not detected in EVs. 

### 2.2. Effects of EVs of HCV Patients on HUVEC

**Figure 7:**
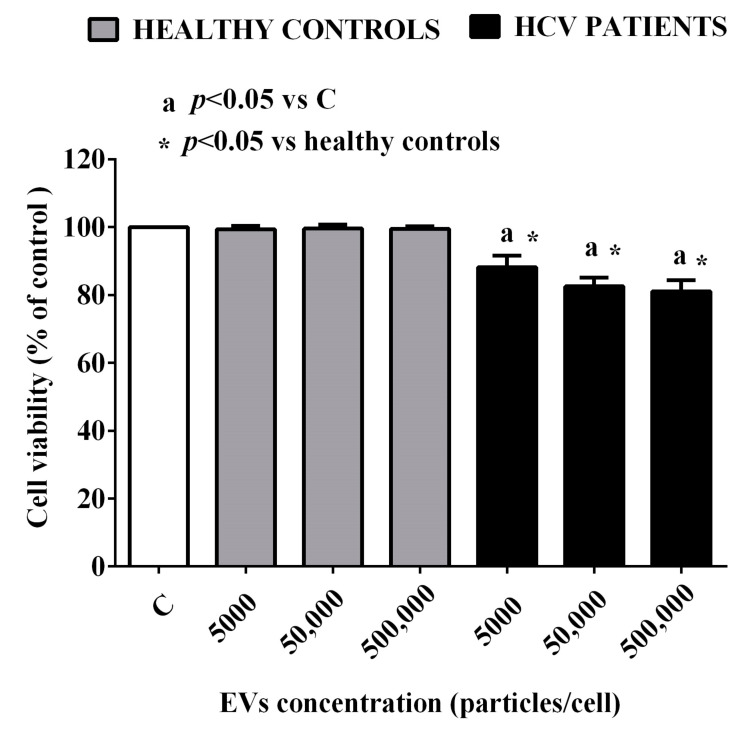
Dose–response effects of EVs from HCV patients and healthy controls on HUVEC viability. The values are the means ± SD of repeated measurements. C: non-treated cells.

As a first step, we performed a dose–response study to analyze the effects of different EV concentrations on HUVEC. In particular, we used 5000 EVs/cell, 50,000 EVs/cell, and 500,000 EVs/cell for 24 h and then we examined the effects on cell viability. As shown in Figure 7, EVs isolated from healthy controls did not cause any effect on HUVEC. Instead, the three concentrations of EVs isolated from HCV patients were able to reduce the cell viability of HUVEC in a similar manner. These results, therefore, indicate that the harmful effect of EVs of HCV patients on HUVEC was not related to their concentrations. For all subsequent experiments, we used a concentration of EVs equal to 50,000 EVs/cell. 

**Figure 8:**
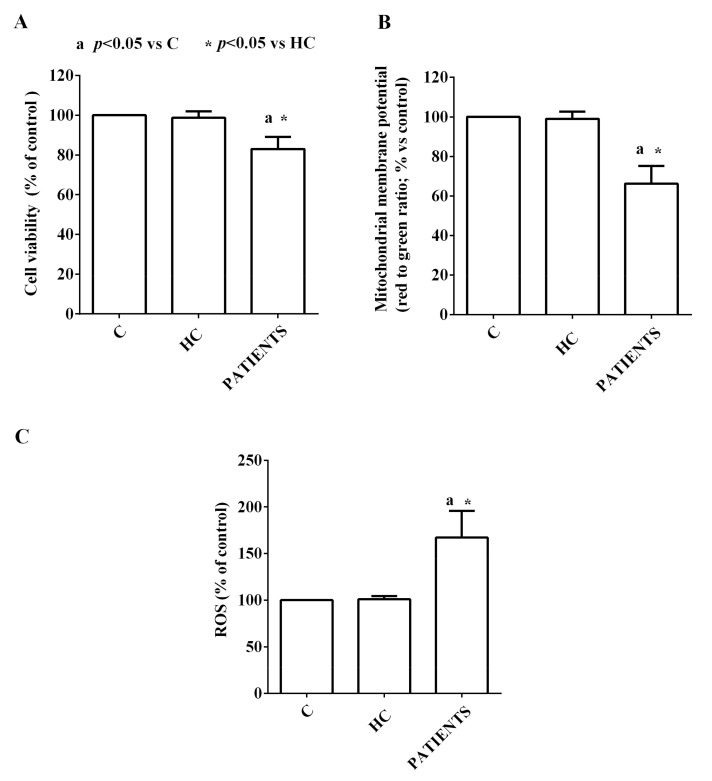
Effects of EVs from HCV patients on viability (**A**), mitochondrial membrane potential (**B**) and reactive oxygen species (ROS) release (**C**) in HUVEC. The values are the means ± SD of repeated measurements. C: non-treated cells. HC: healthy controls.

Treatment of HUVEC with EVs from HCV patients induced deleterious effects in terms of viability, mitochondrial damage, and increased oxidative stress, as shown in Figure 8A–C.

**Figure 9:**
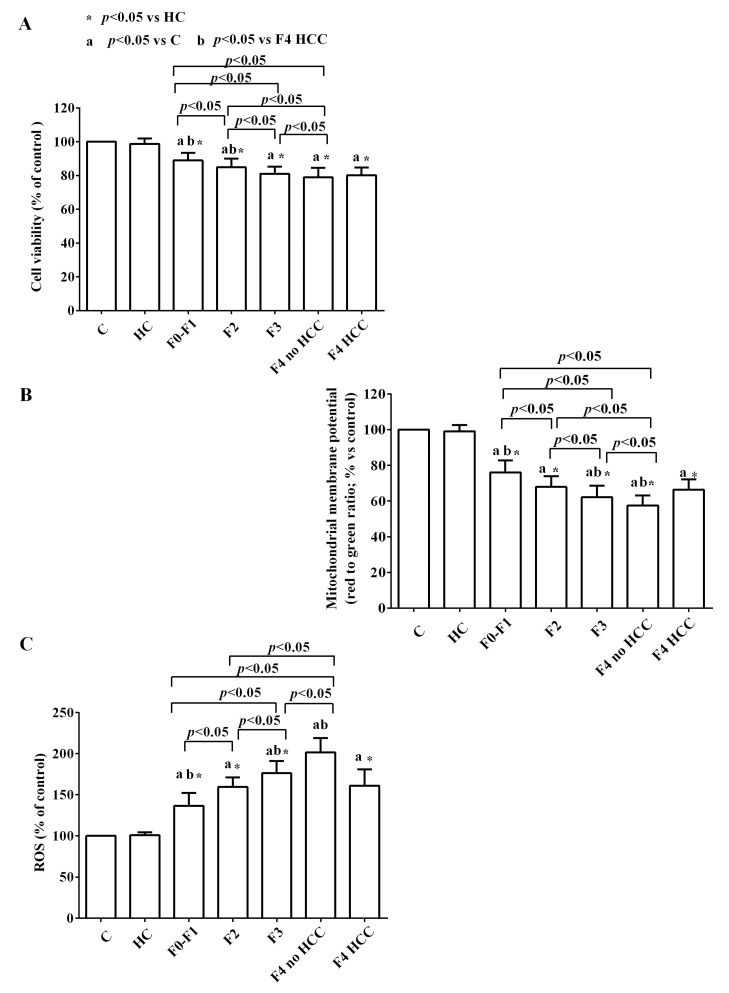
Effects of EVs from HCV patients from F1 to F4 without (F4 no HCC) and with HCV-related HCC (F4 HCC) on viability (**A**), mitochondrial membrane potential (**B**) and reactive oxygen species (ROS) release (**C**) in HUVEC. The values are the means ± SD of repeated measurements. C: non-treated cells. HC: healthy controls. HCC: hepatocarcinoma. F1, F2, F3, and F4: METAVIR HCV stages. No HCC: cirrhosis without HCC. HCC: cirrhosis with HCC. Square brackets indicate significance between groups.

In addition, the EVs isolated from patients with different stages of HCV disease reduced the viability and the mitochondrial membrane potential of HUVEC in a manner related to the stage of the disease itself. More in detail, the highest effect was observed in stage F4 without HCC (Figure 9A,B).

In the same way, we observed an increase in ROS release from HUVEC, which was progressively higher when using EVs isolated from patients from stage F1 to stage F4 without HCC (Figure 9C). In the case of HCV-related cirrhotic HCC patients, the effects of EVs on cell viability were similar to those caused by EVs of the F4 stage without HCC; whereas, as regarding mitochondrial membrane potential and ROS release, the effects were similar to those observed in the F2 stage.

The use of inhibitors of NLRP3, PI3k, and AMPK highlighted the role of the above signaling pathways in the genesis of the effects of EVs in HUVEC. Furthermore, the different involvement in the damage of HUVEC elicited by EVs isolated from HCC patients compared to that observed with EVs isolated from patients with lower stages of disease was highlighted.

**Figure 10:**
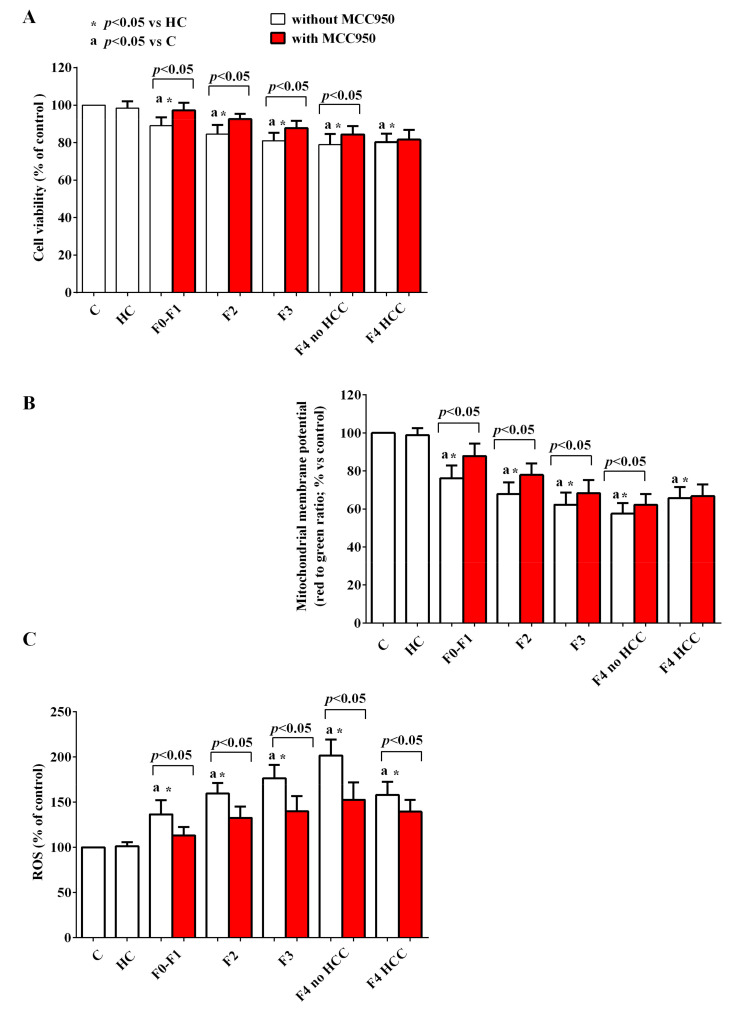
Effects of EVs from HCV patients on viability (**A**), mitochondrial membrane potential (**B**), and reactive oxygen species (ROS) release (**C**) in HUVEC in the absence/presence of the NLRP3 inhibitor, MCC950. The values are the means ± SD of repeated measurements. Square brackets indicate significance within each group before and after the inhibitor.

**Figure 11:**
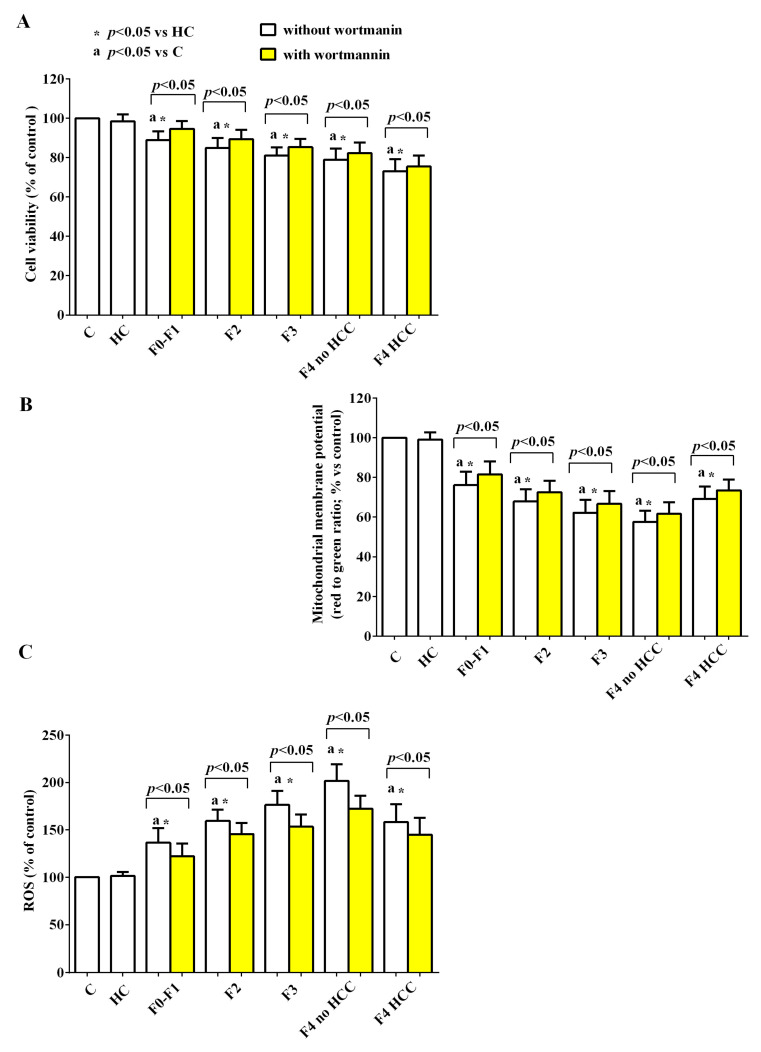
Effects of EVs from HCV patients on viability (**A**), mitochondrial membrane potential (**B**), and reactive oxygen species (ROS) release (**C**) in HUVEC in the absence/presence of the PI3k inhibitor, wortmannin. The values are the means ± SD of repeated measurements. The layout is as in previous figures. Square brackets indicate significance within each group before and after the inhibitor.

**Figure 12:**
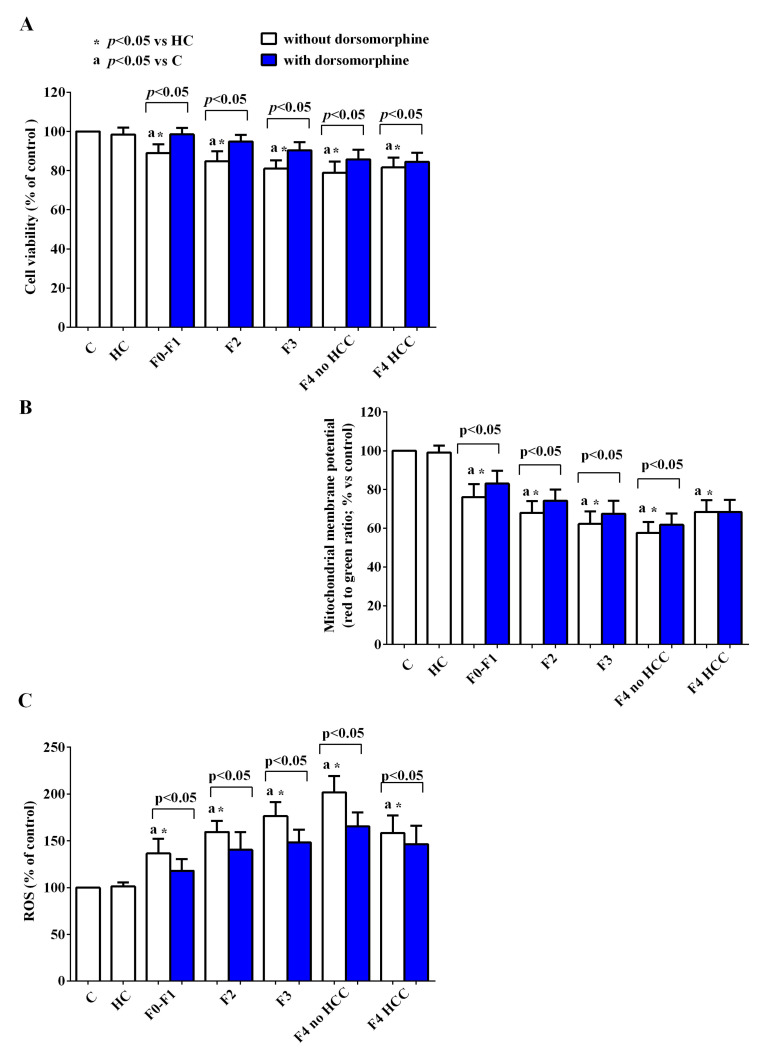
Effects of EVs from HCV patients on viability (**A**), mitochondrial membrane potential (**B**) and reactive oxygen species (ROS) release (**C**) in HUVEC in the absence/presence of the AMPK inhibitor, dorsomorphine. The values are the means ± SD of repeated measurements. The layout is as in previous figures. Square brackets indicate significance within each group before and after the inhibitor.

Indeed, the pretreatment with MCC950 (NLRP3 blocker), wortmannin (PI3k blocker), and dorsomorphine (AMPK blocker) was able to counteract the reduction of cell viability and mitochondrial membrane potential (Figure 10A,B, Figure 11A,B and Figure 12A,B) and the increased ROS release by HUVEC (Figure 10C, Figure 11C and Figure 12C), in particular in HCV subjects with stages of disease from F1 to F4 without HCC. As regards HCV-related HCC patients, the NLRP3 blocker MCC950 was only able to reduce the ROS release (Figure 10C). The use of the PI3k blocker wortmannin and of the AMPK blocker dorsomorphine contrasted the effects of EVs to a significantly reduced extent compared to what was observed with the lower stages of the disease (Figure 11 and Figure 12).

## 3. Discussion

The results of this study are, to the best of our knowledge, the first to highlight the presence in the plasma of HCV patients of a pattern of endothelium-lymphocytes-platelet derived EVs, capable of inducing damage to the endothelium. 

These data could represent a possible pathogenic mechanism underlying the increased occurrence of CVD in chronic hepatitis, one of the main extrahepatic manifestations of this disease, and fit well with the latest evidence about this issue. Indeed, major adverse cardiovascular events, such as coronary artery disease, heart failure, stroke, and peripheral artery disease, have been recently associated with HCV infection [2,30], which would act as an independent risk factor for subclinical and clinical CVD and higher cardiovascular mortality [31,32]. The impact of HCV-related CVD has been estimated to be 1.5 (95% CI: 0.9–2.1) million disability-adjusted life years. Moreover, the majority of people affected are between 55 and 75 years old, which highlights a possible earlier development of CVD in patients with HCV in comparison with non-infected subjects [33]. 

The increased risk of CVD associated with HCV infection may be related to a direct action of the virus, which is able to activate the atherosclerotic process, by causing endothelial dysfunction and vascular smooth muscle cell proliferation and migration [3,34]. Among other various proposed pathophysiological mechanisms for the onset of CVD, which would rather be more related to indirect actions of HCV, systemic inflammation could play a major role with its associated increased release of cytokines such as Interleukin (IL)-1β, IL-6 and Tumor Necrosis Factor (TNF)-α, [35]. Additionally, HCV could induce insulin resistance and type 2 diabetes mellitus, oxidative stress, and fatty liver, all of which could contribute to the development of vascular damage [36]. It is important to underline that the virus-related indirect actions could represent a potential pathogenic mechanism for the onset of CVD even after HCV infection eradication.

In all of the above conditions, the circulating EVs—which have a well-known direct contribution to endothelial pathology as demonstrated by numerous studies showing their pro-inflammatory and vascular damage effects—could play a trigger role [37,38]. Besides hepatitis C, these considerations also apply to other diseases characterized by inflammation and oxidative stress. Hence, infectious agents could induce host cells to release EVs, which, in turn, could provide a long-lasting feedback loop to either exacerbate the infection, local and systemic inflammation, and endothelial dysfunction also at distant sites [24,39], suggesting a mechanistic role of EVs at the intersection of inflammatory processes and CVD [24]. Coming back to the specific context of hepatitis C, this could be crucial not only as regards the well-known damage of EVs on the liver—primarily due to their role in mediating immune response evasion and progressive fibrosis development—[40,41], but also concerning some of the aforementioned extrahepatic manifestations most linked to atherosclerosis and/or coagulation activation, although this still deserves further confirmation at least in patients with mild to moderate liver disease [42,43,44]. Furthermore, this could be an explanation for why the detrimental effect induced by HCV infection can persist over time well after the end of the acute phase or viral eradication [19,45].

The characterization of the EVs that we performed in HCV patients through NanoSight, MACSPlex, and FACS allowed us to highlight features of the EVs potentially correlated with their pathogenic role. First of all, in our study, we found that EVs from HCV-positive subjects were larger than those from healthy controls. In this context, it is to note that there is a growing interest in measuring the concentration of EVs in a sample together with their size distribution. For example, EVs size may be used to infer EVs type (e.g., exosomes, microvesicles, and apoptotic bodies), although the relationship between EVs size and type is less defined than suggested. Moreover, a number of studies recently reported EV size as a possible new biomarker in the context of various inflammatory and malignant diseases. Focusing on liver pathology, our data are in agreement with many other studies showing a larger average EVs in different liver diseases, such as non-alcoholic and alcoholic steatohepatitis or primary sclerosing cholangitis [46]. However, concerning viral hepatitis, the literature evidence is somewhat less solid. In HCV infection, most authors found an average larger EVs than in non-infected controls [24] when addressing issues such as viral disease diagnosis, liver inflammatory activity assessment, or fibrosis detection [39,47] but this finding was not confirmed by others [48,49]. The same applies to chronic viral hepatitis B, where both large [50] and small EVs [51,52] were reported. In any case, it is noteworthy that sizes rather than the concentrations of EVs were found to be correlated with the prognosis of such different conditions as acute liver failure [25], cirrhosis [26], and/or HCC [27], providing evidence for their previously unrecognized role as biomarkers also in this specific context.

In addition, in our study, we found an increased expression of markers of lymphocyte/endothelial derivation (such as CD105, CD62e, CD62p, CD3, CD4, CD8, CD14, CD19, CD29, and CD69) in EVs isolated from HCV patients versus healthy controls. In this context, it is well known that EVs released by activated leukocytes and endothelial cells can promote inflammatory infiltration and the activation of atherogenesis [53]. For this reason, they could be involved also in HCV-related endothelial dysfunction and CVD. Moreover, it is to note that increased levels of CD62e+ EVs have been found in patients with coronary artery disease and have been considered good markers of endothelial activation and evolution from mild to severe injury [54]. On the other hand, CD105, also known as endoglin, has been considered a good marker of endothelial dysfunction in CVD [55] and has been found to be correlated with histological and serum markers of hepatic fibrosis in HCV carriers [56]. 

As far as platelet-derived markers of EVs are concerned, we found an increased expression versus healthy controls only for CD62p, while for the other markers, we could not find any differences. In this regard, however, it should be emphasized that greater involvement of the platelet fraction has so far been highlighted mainly in subjects affected by advanced fibrosis or HCC. In fact, in those patients, platelet-derived soluble mediators such as thromboxane A2, TNF-β, and vesicles containing genetic material could promote tumor progression, angiogenesis, and metastasis [57,58]. In our study, we focused on the analysis of EVs characterization in a population of HCV subjects with all disease stages (including, but not limited to, HCC). Further insight into the nature of EVs centered on HCV-related HCC could thus be the subject of subsequent analyses.

In order to evaluate the possible mechanisms of endothelial damage induced by EVs, we used the latter to stimulate HUVEC. It is noted that also the data obtained in vitro confirmed that the concentrations of EVs were not triggers to induce HUVEC damage. Indeed, we could not find an augmentation in the harmful effects played by EVs in the range from 5000 to 500,000 particles/cell.

Moreover, our results showed that those vesicles were able to induce endothelial dysfunction both through an alteration of the mitochondrial activity and increased oxidative stress. These data about the harmful effects of EVs from non-healthy subjects are in agreement with those obtained by other groups which demonstrated that the EVs isolated from patients with various diseases—such as atherosclerosis, acute coronary syndrome, or chronic inflammatory diseases—can induce endothelial dysfunction in various in vitro and ex vivo models [59,60], probably due to common pathophysiological mechanisms including impaired vasorelaxation and induction of vascular inflammation through increased levels of adhesion molecules, ROS, and proinflammatory cytokines [61]. The use of the MTT and JC-1 assay, which are widely adopted to examine cell viability and mitochondrial membrane potential [62], allowed us to highlight the aforementioned harmful effects of EVs. Indeed, both cell viability and mitochondrial membrane potential decreased in HUVEC. In addition, ROS release, which was evaluated through the DCFDA assay as previously performed in the same or other cell lines, was found to be increased. It is to note that all the observed effects were greater as progressing from METAVIR stage F1 to F4.

As above reported, it is widely accepted that endothelial activation and dysfunction could act as important contributors to atherosclerosis and CVD. In this way, our findings could add information about the pathophysiologic mechanisms at the basis of the onset of CVD in HCV patients. In this regard, a key role could be played precisely by the release of EVs, which could act as trigger factors and may continue to play a deleterious role even after the eradication of the infection [63]. In particular, the changes in mitochondrial function, as shown by the reduction of mitochondrial membrane potential of HUVEC, could represent the starting point of a series of events, which finally would lead to CVD. Hence, the abnormal morphology and dysfunction of mitochondria have been proven as the principal mechanisms in the pathogenesis of CVD, such as atherosclerosis, hypertension, heart failure, or myocardial infarction [64]. In particular, the loss of mitochondrial membrane potential could lead to endothelial dysfunction also through increased oxidative stress [65]. 

Moreover, the results obtained in the presence of specific inhibitors highlighted the role played by NLRP3 inflammasome, AMPK and Akt in the effects of EVs on HUVEC. This observation was especially true for HCV patients of any stage as long as they were without HCC. Indeed, in the presence of various inhibitors of the above pathways (i.e., MCC950, dorsomorphine, and wortmannin) both the reduction of cell viability/mitochondrial membrane potential and the increased ROS release caused by their EVs were counteracted. Quite surprisingly, however, the same inhibitors either did not reverse the effects of EVs isolated from cirrhotic HCC patients (as in the cases of MCC950 on cell viability and mitochondrial membrane potential and of dorsomorphine on mitochondrial membrane potential), or only slightly reduced the damage of HUVEC, suggesting that, when neoplastic foci have already developed, other multiple intracellular signaling pathways are also involved, as shown by others [66,67]. 

The findings we obtained with EVs from HCV subjects are not surprising if we consider that the aforementioned pathways are implicated not only in HCV infection and subsequent liver chronic disease but also in the secondary endothelial damage correlated with the genesis of CVD [20,68]. Moreover, Akt and AMPK have been reported as downstream factors implicated in the effects of EVs or other extracellular mediators on cardiovascular cells [69]. 

Our study has several limitations. The sample size is small, although it should be borne in mind that the techniques used are expensive and difficult to apply to large numbers. On the same line, the pattern of circulating EVs could have been implemented by studying proteomic and transcriptomic expression profiles, specifically in relationship to HCC. In addition, the cellular effects of EVs could have been more deeply analyzed to expand knowledge about mitochondria dysfunction and oxidative stress.

## 4. Materials and Methods

### 4.1. Patients and Healthy Controls

This study included 65 anti-HCV antibodies and HCV-RNA positive patients aged ≥18 years willing to donate a blood sample, whose main characteristics are reported in Table 1. They were selected from a larger group evaluated at a single liver clinic of an academic hospital by one of us (C.S.). Inclusion criteria were (a) medical records containing thorough clinical and laboratory information; (b) a valid baseline transient elastography examination performed within 30 days from the blood sample collection date. Exclusion criteria were (a) history of current or past excessive alcohol consumption (>20 g/d for women, >30 g/d for men); (b) coinfection by hepatitis B and/or human immunodeficiency virus (HIV); (c) autoimmune liver diseases; (d) current anti-HCV treatment; (e) current or past treatments for HCC. The recruitment period was from 30 June 2015 to 30 June 2022. The study design was an observational non-consecutive retrospective case series. Per protocol, it was decided to have a uniform representation of all stages of liver disease.

A group of 5 healthy controls was also recruited, with demographic characteristics comparable to the patient group: 4 males (80%) with median age 63.1 (58.9–64.2) years and BMI 25.1 (23.8–26.8) kg/m^2^.

All subjects gave written informed consent to their participation in the study, which was conducted in strict adherence to the principles of the Declaration of Helsinki of 1975, as revised in 2000. The study protocol was approved by the institutional ethical committee (Comitato Etico Interaziendale Novara, IRB code CE033/2023, https://comitatoetico.maggioreosp.novara.it/IRB, accessed on 1 June 2023).

### 4.2. Blood Sample Collection

In each patient and healthy control, blood samples were taken at 9 am in fasting conditions by using BD Vacutainer tubes (sodium heparin as anticoagulant). Each sample was forthwith centrifuged for 10 min through a centrifuge model 5702 with rotor A-4-38 (Eppendorf SE; Hamburg, Germany) working at 3100 rpm, 4 °C. The plasma was then aliquoted into 6 tubes and was used for the quantification of the most common laboratory analytes according to current standards of clinical practice and for the EVs isolation and the execution of the in vitro experiments. The plasma samples, which were used for the EVs isolation and for the in vitro experiments, were stored at −80 °C at the Physiology laboratory, Università del Piemonte Orientale, in Novara. Plasma samples were always handled in pseudonymized conditions.

### 4.3. EVs Isolation

EVs were isolated by ultracentrifugation (Beckman Coulter Optima™ LE-80K; Beckman Coulter; Indianapolis, IN, USA). To do this, 2 mL of plasma sample were diluted with phosphate buffer saline (PBS) until reaching the final volume of the 4 mL tubes (Beckman Coulter, Milan, Italy). The tubes were then placed into the SW 60 Ti swinging-bucket rotor (Beckeman Coulter) and the ultracentrifuge was set as follows: 100,000× *g*, 4 °C, 2 h, as previously performed [70].

Afterward, the supernatant was removed and the pellet was resuspended in 1 mL fetal bovine serum (FBS) free Dulbecco’s Modified Eagle Medium (DMEM), and then stored at −80 °C.

### 4.4. EVs Characterization

Isolated EVs were diluted 1:200 in a 0.1 µm filtered physiological solution (sodium chloride 0.9%; B. Braun; Milan, Italy) and analyzed by NanoSight (NS300; Malvern Panalytical; Malvern, UK) equipped with the Nanoparticle Tracking Analysis (NTA) and NTA 3.2 Analytical Software Update. A syringe pump flow of 30 was applied for each sample. Three videos of 60 s each were recorded and analyzed, calculating an average number of EV sizes and concentrations (particles/mL). 

Furthermore, the fluorescence-activated cell sorting (FACS) was used to examine CD41a and CD62e EVs surface markers, which was executed by means of Attune™ NxT flow cytometer (Thermo Fisher Scientific; Waltham, MA, USA). Before FACS measurements, EVs were diluted 1:100 with PBS in 1.5 mL tubes, which was followed by the addition of a fluorescently tagged antibody against a specific surface marker in a ratio 1:1 with the EVs. After the addition of the antibody, the plate was incubated for 1 h at 4 °C protected from light. EVs deriving from each patient/healthy control were plated and analyzed at least in triplicate. Comparisons were made vs non-treated cells (set as 1). CD41a was tagged with fluorescein isothiocyanate (FITC; BD Biosciences; San Jose, CA, USA) whereas CD62e was tagged with phycoerythrin (PE; BD Bioscience). As a control, EVs deriving from each patient were also stained with FITC and PE mouse Isotypic IgG (BD Biosciences).

#### MACSPlex Exosome Kit Analysis

Human MACSPlex Exosome kit (Miltenyi Biotec; San Jose, CA, USA) was used to analyze the expression on EVs surface of 37 exosomal surface epitopes. For this analysis, 1 × 10^9^ EVs (EV amount estimated based on NTA quantification analysis) were diluted in MACSPlex Buffer (MPB) until a final volume of 120 µL. After the addition of 15 µL of MACSPlex Exosome Capture beads, each sample was incubated overnight at 4 °C under gentle agitation and protected from light. Thereafter, EV-bead complexes were washed adding 500 µL of MACSPLEX buffer and centrifuged at 3000× *g* for 5 min at room temperature. A total of 500 µL of supernatant were then aspirated, and a mixture of anti-CD9, anti-CD63 and anti-CD81 (5 µL each) APC-conjugated antibodies was added to each sample. After 1 h incubation under gentle agitation at room temperature, 500 µL of MACSPlex buffer was added, followed by a centrifugation at 3000× *g* for 5 min. Afterwards, another washing was performed. Finally, after centrifugation, 350 µL of supernatant was removed, and the remaining part of the volume was used to resuspend the pellet. Flow cytometry analysis was performed using FACS Celesta (BD Biosciences; Franklin Lakes, NJ, USA). Background values of MACSPlex buffer and the isotype controls (recombinant engineered antibody (REA) or mouse IgG) were subtracted from each sample. The values were then normalized to the mean median fluorescence intensity (MFI) of the tetraspanins (CD9, CD63, CD81) and expressed as percentages.

### 4.5. Human Umbilical Vein Vascular Endothelial Cells (HUVEC)

HUVEC were purchased from ATCC (Manassas, VA, USA) (catalog no. CRL-1730™) and were maintained in Kaighn’s Modification of Ham’s F-12 Medium (F-12K Medium; ATCC; catalog no. 30-2004™), containing 2 mM L-glutamine (Euroclone S.p.A, Milan, Italy), 1500 mg/L sodium bicarbonate (Euroclone), and supplemented with 0.1 mg/mL heparin (Merck KGaA, Darmstadt, Germany), 1% penicillin, 1% streptomycin, and 10% FBS (Euroclone).

These cells were stimulated for 24 h with EVs of HCV patients and healthy controls. In the first phase of the study, we performed a dose–response analysis of the effects of EVs on cell viability (at 5000 EVs/cell, 50,000 EVs/cell and 500,000 EVs/cell) by using the MTT assay. Thereafter, we examined the effects of EVs (50,000 EVs/cell) on HUVEC mitochondrial membrane potential (i.e., JC1 assay) and ROS release (i.e., DCFDA assay).

Moreover, we evaluated the role of inflammasome NLR family pyrin domain containing 3 (NLRP3), phosphoinositide 3-kinase (PI3k), and AMP-activated protein kinase (AMPK) in the effects of EVs of HCV patients on HUVEC. This was conducted by repeating the stimulation with EVs in the presence of various inhibitors of the above pathways (MCC950, wortmannin and dorsomorphine, respectively; 1 nM, 30 min stimulation for all three reagents [70,71]. The experiments were performed in triplicate and repeated at least three times by using different pools of HUVEC.

### 4.6. MTT Assay

Cell viability of HUVEC was investigated by using the 1% 3-[4,5-dimethylthiazol-2-yl]-2,5-diphenyl tetrazolium bromide (MTT assay; Cayman Chemical, Ann Arbor, MI, USA), as previously performed in similar or other cellular models [71,72,73,74].

The 10% of the MTT solution was prepared by dissolving 50 mg of the MTT reagent (3-(4,5-dimethylthiazol-2-yl)-2,5-diphenyl tetrazolium bromide) in 10 mL of PBS (pH 7.4) and kept stored at 4 °C protected from the light. Following the EVs stimulation of HUVEC (10,000 cells/well in 96 well plates) for 24 h at the selected concentration, the media was removed and 100 µL of the MTT solution diluted in DMEM high glucose *w*/*o* phenol red, supplemented with 2 mM L-glutamine, and 1% penicillin-streptomycin (P/S) were added to each well. Thereafter, the plate was incubated at 37 °C for 2 h.

Once the reaction had occurred, the supernatant was removed and the formazan crystals formed in each well were dissolved with 100 µL of dimethyl sulfoxide (DMSO; Sigma, Milan, Italy). Cell viability was finally determined by measuring the absorbance through a spectrophotometer (VICTOR™ X Multilabel Plate Reader; PerkinElmer; Waltham, MA, USA) with a wavelength of 570 nm. Cell viability was calculated by setting control cells (untreated cells) as 100%.

### 4.7. JC-1 Assay

Mitochondrial membrane potential (ΔψM) is an important parameter of the mitochondrial function used as an indicator of cell health, as previously tested in similar cellular models [61,62,63,64,65,66,67,68,69,70,71].

In order to examine the mitochondrial membrane potential, the medium of HUVEC stimulated with EVs (as performed for MTT assay) was removed and cells were incubated with 5,51,6,61-tetrachloro-1,11,3,31tetraethylbenzimidazolyl carbocyanine iodide (JC-1) staining solution diluted in Assay Buffer 1X (Cayman Chemical; Ann Arbor, MI, USA) for 20 min at 37 °C, following the manufacturer’s instructions. After incubation, HUVEC were washed twice with the Assay buffer 1X and then 100 µL/well of it was added for the final reading. The mitochondrial membrane potential was determined by measuring the red (excitation 535 nm/emission 595 nm) and green (excitation 485 nm/emission 535 nm) fluorescence through a spectrophotometer (VICTOR™ X Multilabel Plate Reader; PerkinElmer). Normalization of the data was executed versus control cells (untreated cells).

### 4.8. DCFDA Assay

The oxidation of 2,7-dichlorodihydrofluorescein diacetate (H2DCFDA) into 2,7-dichlorodihydrofluorescein (DCF) was used to assess ROS generation, following the manufacturer’s instructions (Abcam; Cambridge, UK), and as previously performed [72,75]. 

After stimulation of HUVEC with EVs, as described for MTT and JC assays, the medium was removed, and staining was performed with 10 μM H2DCFDA for 20 min at 37 °C. The fluorescence intensity of DCF was measured at an excitation and emission wavelength of 485 and 530 nm, respectively, by using a spectrophotometer (VICTOR™ X Multilabel Plate Reader; PerkinElmer). Results were expressed as DCF fluorescence intensity, which was proportional to the amount of intracellular ROS. The data were normalized versus control cells (untreated cells).

### 4.9. Statistical Analysis

Statistical analysis and graphs were executed by using GraphPad Prism version 9.0.0 (GraphPad Software; San Diego, CA, USA). 

As regarding patients, data are presented as medians (interquartile range) for continuous variables and as frequencies (%) for categorical variables.

As regarding the results on EVs and of the in vitro studies, data were checked for normality before statistical analysis. The differences between two groups were assessed through Kruskal–Wallis and Mann–Whitney U-tests, as appropriate. All data are presented as means ± standard deviation (SD) of repeated measurements. 

*p* values were considered significant when equal to or below 0.05 and were two-tailed.

## 5. Conclusions

The results obtained in this study highlight the role of circulating EVs in HCV patients in inducing endothelial damage that may project its effects even after the eradication of the infection. In an era in which eliminating HCV infection is feasible in the vast majority of patients, our study, while postulating novel mechanisms of viral-induced damage, provides further support to the concept that treatment of HCV infection is a way to prevent both hepatic and extra-hepatic complications.

## Figures and Tables

**Table 1:** Main demographic and clinical features of the study population (*n* = 65). Fibrosis staging is according to METAVIR classification. Data are presented as medians (interquartile range) for continuous variables and as frequencies (%) for categorical variables.

Feature	Value
Male sex, *n*	54 (84)
Age, years	67.8 (60.1–77.1)
Body mass index (BMI), kg/m^2^	26.1 (23.8–27.2)
HCV genotype, *n* for 1a, 1b, 2, 3, 4	3 (5), 47 (76), 9 (14), 3 (5), 0 (0)
HCV RNA, ×10^3^ IU/mL	1213 (296–2465)
Status of previous PEG-IFN treatment, *n* for naïve, experienced	45 (69), 20 (31)
Diabetes mellitus, *n*	50 (77)
Hypertension, *n*	28 (43)
Metabolic syndrome *, *n*	49 (75)
Baseline transient elastography, kPa	11.0 (7.4–15.6)
Stage of liver disease:	
No/mild fibrosis(F1) *n*	13 (20)
Moderate fibrosis (F2), *n*	13 (20)
Severe fibrosis (F3), *n*	13 (20)
Cirrhosis (F4) without HCC, *n*	13 (20)
Cirrhosis (F4) and HCC, *n*	13 (20)
Child-Pugh class, *n* for A, B, C **	22 (80), 4 (20), 0 (0)
MELD score **	8.0 (7.0–9.8)
BCLC stage, *n* for 0, A, B, C ***	1 (7), 4 (31), 4 (31), 4 (31)
ALT, U/L	69 (37–97)
Total bilirubin, mg/dL	1.0 (0.7–1.2)
International Normalized Ratio, Units	1.1 (1.0–1.2)
Platelets, ×10^9^/L	167 (122–212)
Creatinine, mg/dL	0.8 (0.7–0.9)
Albumin, g/L	40 (39–44)
Plasma total cholesterol, mg/dL	160 (135–167)
Plasma triglycerides, mg/dL	79 (54–94)

HCV: hepatitis C virus; PEG-IFN: pegylated interferon-based; HCC: hepatocellular carcinoma; MELD: Model for end-stage liver disease; BCLC: Barcelona Clinic Liver Cancer; ALT: alanine aminotransferase; NCEP: National Cholesterol Education Program; ATP III: Adult Treatment Panel III. * according to NCEP ATP III classification; ** for cirrhotic patients; *** for HCC patients.

## Data Availability

Data are available on reasonable request.
